# Involvement of TSC genes and differential expression of other members of the mTOR signaling pathway in oral squamous cell carcinoma

**DOI:** 10.1186/1471-2407-8-163

**Published:** 2008-06-06

**Authors:** Sanjukta Chakraborty, SM Azeem Mohiyuddin, KS Gopinath, Arun Kumar

**Affiliations:** 1 Department of Molecular Reproduction Development and Genetics, Indian Institute of Science, Bangalore, India; 2 RL Jalappa Hospital and Research Centre, Kolar, India; 3 Bangalore Institute of Oncology, Bangalore, India

## Abstract

**Background:**

Despite extensive research, the five-year survival rate of oral squamous cell carcinoma (OSCC) patients has not improved. Effective treatment of OSCC requires the identification of molecular targets and signaling pathways to design appropriate therapeutic strategies. Several genes from the mTOR signaling pathway are known to be dysregulated in a wide spectrum of cancers. However, not much is known about the involvement of this pathway in tumorigenesis of OSCC. We therefore investigated the role of the tumor suppressor genes, *TSC1 *and *TSC2*, and other members of this pathway in tumorigenesis of OSCC.

**Methods:**

Expression of genes at the RNA and protein levels was examined by semi-quantitative RT-PCR and western blot analyses, respectively. Loss of heterozygosity was studied using matched blood and tumor DNA samples and microsatellite markers from the *TSC1*, *TSC2 *and *PTEN *candidate regions. The effect of promoter methylation on TSC gene expression was studied by treating cells with methyltransferase inhibitor 5-azacytidine. Methylation status of the *TSC2 *promoter in tissue samples was examined by combined bisulfite restriction analysis (COBRA).

**Results:**

The semi-quantitative RT-PCR analysis showed downregulation of *TSC1*, *TSC2*, *EIF4EBP1 *and *PTEN*, and upregulation of *PIK3C2A*, *AKT1*, *PDPK1*, *RHEB*, *FRAP1*, *RPS6KB1*, *EIF4E *and *RPS6 *in tumors. A similar observation was made for AKT1 and RPS6KB1 expression in tumors at the protein level. Investigation of the mechanism of downregulation of TSC genes identified LOH in 36.96% and 39.13% of the tumors at the TSC1 and TSC2 loci, respectively. No mutation was found in TSC genes. A low LOH rate of 13% was observed at the PTEN locus. Treatment of an OSCC cell line with the methyltransferase inhibitor 5-azacytidine showed a significant increase in the expression of TSC genes, suggesting methylation of their promoters. However, the 5-azacytidine treatment of non-OSCC HeLa cells showed a significant increase in the expression of the *TSC2 *gene only. In order to confirm the results in patient tumor samples, the methylation status of the *TSC2 *gene promoter was examined by COBRA. The results suggested promoter hypermethylation as an important mechanism for its downregulation. No correlation was found between the presence or absence of LOH at the TSC1 and TSC2 loci in 50 primary tumors to their clinicopathological variables such as age, sex, T classification, stage, grade, histology, tobacco habits and lymph node metastasis.

**Conclusion:**

Our study suggests the involvement of TSC genes and other members of the mTOR signaling pathway in the pathogenesis of OSCC. LOH and promoter methylation are two important mechanisms for downregulation of TSC genes. We suggest that known inhibitors of this pathway could be evaluated for the treatment of OSCC.

## Background

Oral squamous cell carcinoma (OSCC) is the sixth most common cancer in the world [1]. In India, it is the leading cancer among males and the third most common malignancy in females [1]. The five-year survival rate for OSCC is the lowest among all major cancers [1]. The etiology of this cancer is multifactorial, with important risk factors being tobacco intake, alcohol consumption and human papilloma virus (HPV).

A thorough understanding of the genetic and epigenetic changes that result in the activation of signaling pathways and provide the cells with a growth advantage during oral tumorigenesis is essential for the development of novel therapeutic strategies. Agents that can inhibit or reverse these changes by targeting molecularly defined pathways should receive increased attention as novel candidates for oral cancer prevention and therapy [2,3]. The molecular interplay between phosphoinositide-3-kinase, catalytic, alpha polypeptide (PIK3CA) and FK506 binding protein 12-rapamycin associated protein 1 (FRAP1) of the mTOR (mammalian target of rapamycin) signaling pathway in the control of cell growth and proliferation has been the subject of much interest among cell biologists [4]. Tuberin, encoded by the tumor suppressor gene tuberous sclerosis 2 (*TSC2*), and its interacting partner hamartin, encoded by another tumor suppressor gene tuberous sclerosis 1 (*TSC1*), have been placed as a complex in the mTOR signaling pathway and negatively regulate the pathway to inhibit mTOR mediated downstream signaling [4]. Several components of the mTOR signaling pathway are known to be dysregulated in a wide spectrum of human cancers [5]. Although some components (*PIK3C2A*, *AKT1*, *PTEN, RPS6 *and *EIF4E*) of this pathway have been implicated in OSCC [6-9], a comprehensive analysis is lacking. Further, very little is known about the roles of TSC tumor suppressor genes in tumorigenesis of OSCC [10]. The main aim of this study was to assess the role of TSC genes and other members of this pathway in the tumorigenesis of OSCC. The results of our study are presented here.

## Methods

### Sample collection

A total of 52 OSCC (oral cancer) samples were ascertained at Bangalore Institute of Oncology, Bangalore. All tumor samples were from the tongue and cheek areas of the mouth. Lesions were situated at the anterior 2/3 of the tongue over the lateral borders, a common site in Indian patients. This study was performed with informed written consent from the patients and approval from the ethics committees of the Bangalore Institute of Oncology and Indian Institute of Science. The specimens were obtained as biopsy or surgical samples from oral cancerous lesions and adjacent normal mucosa (taken from the farthest margin of the surgical resection). The patients had not been treated at the time of biopsy/surgery. The clinicopathological data for 52 patients is given in Table 1. Tumors were classified according to TNM (Tumor, Node and Metastasis) criteria [11]. Peripheral blood samples were also collected in EDTA-Vacutainer (Beckton-Dickinson, Franklin Lakes, NJ) tubes from 52 patients.

**Table 1:** Clinicopathological features of patients included in the study.

**Characteristics**	**No. of patients **(n = 52)
**Median age/range**	50 yrs/32–70 yrs
**Gender**	
Males	14 (26.92%)
Females	38 (73.08%)
**Tumor classification**	
T1	2 (3.85%)
T2	8 (15.38%)
T3	14 (26.92%)
T4	26 (50%)
Epithelial dysplasia	2 (3.85%)
**Tobacco use**	
Tobacco positive	50 (96.15%)
Tobacco negative	2 (3.85%)
**Treatment**	
Surgery	50 (96.15%)
No surgery	2 (3.85%)
Radiotherapy	47 (90.38%)
Chemotherapy	5 (9.62%)
**Lymph node**	
Positive	32 (61.54%)
Negative	16 (30.77%)
Unknown	4 (7.69%)

### Cell culture

Three oral cancer cell lines (SCC 131, SCC 104 and KB) and four other cell lines (HeLa, HepG2, A549 and HEK-293T) were used. SCC 131 and SCC 104 cell lines were a kind gift from Dr. Susanne M. Gollin (University of Pittsburgh, Pittsburgh, PA). Cell lines were maintained either in Minimum Essential Medium with Earle's salt and l-glutamine or in Dulbecco's modified Eagle's medium (Sigma-Aldrich, St. Louis, MO).

### Genomic DNA isolation

Genomic DNA was isolated from peripheral blood and tumor samples using a DNA isolation kit (Roche Diagnostics™, Mannheim, Germany).

### Semi-quantitative RT-PCR

Total RNA was isolated from 16 paired normal and tumor samples using the TRI REAGENT™ (Sigma-Aldrich, St. Louis, MO). cDNA was synthesized from 1 μg total RNA from each sample using random hexamers and the Revertaid™ H Minus First Strand cDNA Synthesis Kit (MBI Fermentas, Burlington, ON, Canada). For RT-PCR, forward and reverse primers were selected from two different exons of genes to rule out the possibility of amplification of contaminating genomic DNA. Primer sequences and PCR conditions are available from the authors upon request. For each gene, the PCR protocol was optimized in order to get the amplification in a linear phase. Glyceraldehyde-3-phosphate dehydrogenase (*GAPDH*) was amplified as a normalizing control. Images of RT-PCR ethidium bromide stained agarose gels were acquired with a Kodak CCD camera and quantification of the bands was performed by densitometric analysis using the Kodak Digital Science Image Station Imaging Software version 3.6.1. Band intensity was expressed as relative absorbance units. Data was expressed in arbitrary units (relative expression) as a ratio of normal/*GAPDH *and tumor/*GAPDH *and plotted using the GraphPad Prism software version 4.00 (GraphPad Prism Software, San Diego, CA). The significance of difference in mRNA levels between normal and tumor samples for a gene was assessed by Student's t-test and the results are expressed as mean ± SEM [12]. A probability value of p < 0.05 was assumed to be significant. PCR amplification for each gene was repeated once. A gene was considered to be upregulated when its mean expression value across 16 tumor samples was significantly higher than the mean expression value across 16 normal tissue samples and vice versa [13]. We defined the cutoff value for determining the upregulation or downregulation of a gene in a tumor sample as ≥ 1.8 fold difference in its expression between normal and tumor samples as described by Arora et al. [3] for differentially expressed genes in oral squamous cell carcinoma.

### Mutation analysis

Mutation screening of the entire coding regions of TSC genes was carried out using PCR-SSCP [14] and DNA sequencing techniques.

### LOH analysis at TSC1, TSC2 and PTEN loci

For LOH studies, matched normal and tumor DNA samples from 50 patients were genotyped using following microsatellite markers: D9S179, D9S1830 and D9S915 for the TSC1 locus; D16S3024, D16S3395 and D16S475 for the TSC2 locus; and D10S215, D10S1765 and D10S541 for the PTEN locus. Microsatellite analysis was performed as described in Kumar et al. [15]. LOH was scored if there was a complete loss of one of the two heterozygous alleles in tumor DNA or a decrease of 50% intensity of one of the two alleles in tumor DNA as compared to the corresponding peripheral blood DNA (allelic imbalance).

### Antibodies and western blot analysis

Rabbit polyclonal antibodies generated against amino acids 488–1016 of TSC1 and amino acids 155–541 of TSC2 were raised in our laboratory. Mouse monoclonal anti-β-actin antibody was purchased from Sigma-Aldrich (St. Louis, MO). Rabbit polyclonal anti-Akt 1/2, anti-p-Akt 1/2/3 (Thr 308) and anti-p-p70S6K1 (Thr 389) antibodies were purchased from Santa Cruz Biotechnology (Santa Cruz, CA). Rabbit polyclonal anti-p70S6K1 antibody was obtained as a kind gift from Dr. I. Juhan-Vague (Marseille, Cedex, France).

For western blot analysis, whole cell lysates were prepared from matched normal and tumor samples as well as cell lines using a standard procedure. Equal amounts of protein (~100 μg/lane) from tumor, normal oral tissue or different cancer cell lines were resolved by SDS-polyacrylamide gel electrophoresis and transferred onto a PVDF membrane. Primary antibody was detected with either HRP-conjugated goat anti-rabbit or goat anti-mouse secondary antibodies (Bangalore Genei™, India). Immunoreactive bands were visualized using the Western Lightning Chemiluminescence Reagent kit (PerkinElmer Life Sciences, Boston, MA) and X-ray films. β-actin was used to see equal protein loading.

### 5-azacytidine treatment of cell lines

SCC 131 and HeLa cells were seeded at a density of 1 × 10^6 ^cells/90 mm dish. After 24 hr, freshly prepared 5-azacytidine (Sigma-Aldrich, St. Louis, MO) was added into the dish to a final concentration of 10 μM. Total RNA was isolated after 2 and 5 days from the start of the treatment. Untreated cells were used as controls. Semi-quantitative RT-PCR was used to assess the expression of *TSC1 *and *TSC2*. *GAPDH *was used as a normalizing control.

### Combined bisulfite restriction analysis

Methylation status of the *TSC2 *gene promoter was examined using combined bisulfite restriction analysis (COBRA) as described by Xiong and Laird [16]. Sodium bisulfite treated DNA was used in PCR amplification using primers designed for the bisulfite treated DNA. Primers were designed using the MethPrimer program [17]. Promoter region of the *TSC2 *gene is reported by Kobayashi et al. [18]. Sodium bisulfite treated DNA was amplified with following *TSC2 *promoter primers: F-5'gggattttagtttgtagtttttattt-3' and R-5'-ccataacttaaaactaaaaaatact-3'. Primers were designed to exclude binding to any CpG dinucleotide to ensure amplification of both methylated and unmethylated forms of DNA. PCR conditions for primers were as follows: an initial denaturation at 95°C for 3 min was followed by 35 cycles of 94°C for 30 sec, 60°C for 45 sec and 68°C for 45 sec with a final extension at 68°C for 5 min. *TSC2 *primer set generated a 571 bp amplicon. A second PCR was carried out using the product of the first amplification as a template to get enough DNA for COBRA. Approximately 500–600 ng of pooled and gel purified PCR product was digested with *Aci *I at 37°C for 6 hr. Digests were resolved in a 2.5% agarose gel and visualized by ethidium bromide staining. There are 18 *Aci *I sites and 65 CpGs in the *TSC2 *promoter. The restriction enzyme *Aci I *recognizes the sequence 5'-GCGG-3'. The cleavage of this sequence will occur only when the C residue in the recognition sequence is methylated. Promoter of *TSC1 *has been reported by Ali et al. [19]. Primers were also designed for the *TSC1 *promoter. However, despite repeated efforts using DNA polymerases from several vendors, we were not successful in amplifying the *TSC1 *promoter after bisulfite treatment.

## Results and discussion

### Downregulation of TSC genes

**Figure 1:**
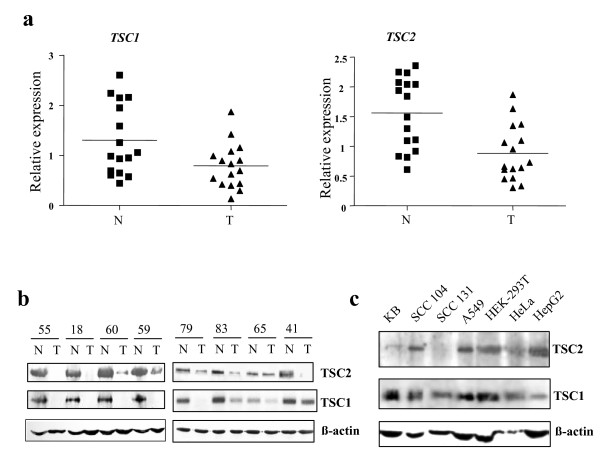
**Expression of TSC genes in oral tumor samples and cell lines.** a) Semi-quantitative RT-PCR analysis of TSC genes in 16 matched normal and tumor samples. Note, downregulation of both genes in tumor samples. Each square or triangle corresponds to data from one sample. Horizontal lines represent mean values of mRNA expression across normal or tumor samples. b) Western blot analysis of TSC1 and TSC2 in eight matched normal and tumor tissues. TSC1 and TSC2 show no expression or downregulation in tumor samples. c) Western blot analysis of TSC1 and TSC2 in three oral cancer (KB, SCC 104 and SCC 131), lung carcinoma (A549), embryonic kidney (HEK-293T), cervical carcinoma (HeLa) and hepatic carcinoma (HepG2) cell lines. Note, the expression of TSC2 is not detectable in the oral cancer cell line SCC 131.

Expression levels of TSC genes were studied by semi-quantitative RT-PCR analysis in a panel of 16 matched normal and tumor samples. The mean expression level of *TSC1 *was significantly lower (1.31 ± 0.18 in normal vs. 0.79 ± 0.11 in tumor, p = 0.0208) in tumor samples (Figure 1a). The mean expression level of *TSC2 *was also significantly lower (1.56 ± 0.15 in normal vs. 0.88 ± 0.12 in tumor, p = 0.0013) in tumor samples (Figure 1a), suggesting the involvement of these genes in the etiology of oral cancer. TSC1 and TSC2 were also downregulated in eight matched normal and tumor samples at the protein level (Figure 1b). We wanted further to determine the expression of both the TSC genes in cell lines. Interestingly, TSC2 did not show a detectable level of expression in an oral cancer cell line SCC 131, whereas it was expressed in two other oral cancer cell lines KB and SCC 104 as well as in A549, HEK-293T, HeLa and HepG2 at the protein level (Figure 1c). However, the TSC2 expression in KB was lower than in other cell lines (Figure 1c). TSC1 was expressed in all the cell lines, albeit at different levels (Figure 1c).

### Mechanisms of downregulation of TSC genes

**Figure 2:**
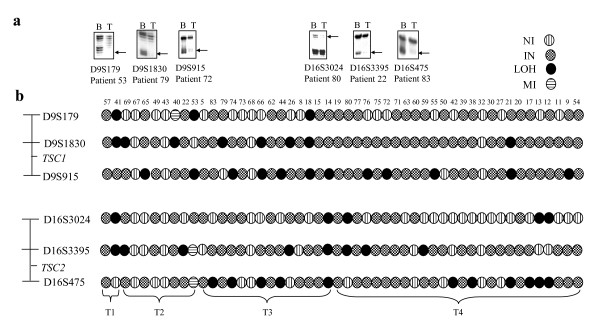
**LOH at TSC loci in 50 matched normal and tumor samples.** a) Representative gel pictures showing LOH for markers from the *TSC1 *and *TSC2 *candidate regions. B and T denote constitutive blood and tumor DNA respectively. Arrows indicate loss or allelic imbalance of the corresponding allele in tumor DNA. b) LOH analysis of 50 matched samples at both the TSC loci. Approximate locations of microsatellite markers with respect to TSC genes are shown on the left. Tumors are grouped according to their T classification (T1–T4). Numbers represent patient numbers. Abbreviations: NI, non-informative; IN, informative; LOH, loss of heterozygosity; and MI, microsatellite instability.

We then sought to determine the mechanism of downregulation of TSC genes in tumor samples. Given the fact that both are tumor suppressors, we hypothesized that their downregulation could be due to inactivating somatic mutations, LOH and/or promoter methylation in tumors. Mutation analysis of the entire coding regions of both the TSC genes did not detect any mutation in a panel of 25 tumor samples. However, four normal population sequence variants (c.965T>C/p.M322T, c.IVS10+51T>A, c.IVS10+27C>G and c.1335A>G/p.E445E) were identified in *TSC1*. Three normal population sequence variants (c.1578C>T/p.S526S, c.IVS14-14C>T and c.2580T>C/p.F860F) were detected in *TSC2*. Interestingly, our analysis of matched peripheral blood and tumor DNA samples from 50 patients showed LOH at both the TSC loci (Figure 2). Of 50 patients, 46/50 patients were constitutionally heterozygous for one or more markers at each of the two TSC loci (Figure 2b). At the TSC1 locus, 17/46 (36.96%) tumors showed an allelic loss for one or more markers. The frequency of LOH at each of the markers analyzed was as follows: 3/29 (10.34%) informative cases for D9S179, 8/45 (17.78%) informative cases for D9S1830 and 12/44 (27.27%) informative cases for D9S915 (Figure 2b). At the TSC2 locus, 18/46 (39.13%) informative cases showed an allelic loss for one or more markers. LOH was found in 5/25 (20%) informative cases for D16S3024, 8/38 (21.05%) informative cases for D16S3395 and 11/37 (29.73%) informative cases for D16S475 (Figure 2b). Nine patients (9/46; 19.57%) had LOH at both the TSC loci (Figure 2b). Microsatellite instability (MI) was found in patient 40 at D9S179 and patient 53 at D16S3395 and D16S475 (Figure 2b). No LOH was found in two ED (epithelial dysplasia) samples at either of the two TSC loci (data not shown).

In spite of the exciting link of TSC genes with the mTOR signaling pathway so widely linked with the onset and progression of tumorigenesis, only few studies have investigated the role of TSC genes in sporadic cancers. Hebert et al. [10] have detected a few mutations in both TSC genes in head and neck tumors from the U.S. patients and SCC cell lines. Although reduced expression of tuberin was found in sporadic astrocytomas, no intragenic mutations were detected in either TSC gene [20]. LOH was seen in astrocytomas, ependymomas, gangliogliomas, glioblastoma multiforme, oligodendrogliomas and pilocytic astrocytomas at both the TSC loci [20]. Parry et al. [21] did not detect any intragenic somatic mutation in TSC genes in sporadic renal cell carcinomas, although LOH was found at both the TSC loci. Somatic mutations in *TSC1 *have been identified in bladder tumors [22]. LOH at the TSC loci has been reported in several other tumor types: lung adenocarcinoma, gall bladder cancer, nasopharyngeal cancer, papillary breast tumors and ovarian serous adenocarcinoma [22,23]. The present study is the first report to describe LOH at both the TSC loci in OSCC. Further, the expression of the *TSC1 *and *TSC2 *genes was found to be significantly downregulated in a majority of oral tumors, both at the RNA and protein levels (Figure 1, Table 2). These results suggest that both the TSC genes act as tumor suppressors in tumorigenesis of OSCC. Our results are in agreement with the studies carried out on both *TSC1 *and *TSC2 *in breast cancer and on *TSC2 *in pancreatic cancer, which have recorded reduced and aberrant expression of these genes [24,25]. Although LOH and somatic mutations are important mechanisms for downregulation of tumor suppressor genes, there are examples, where LOH without somatic mutations has been found to be responsible for the downregulation of these genes. For example, LOH without somatic mutations in TSC genes was found to be an important mechanism for downregulation of these genes in sporadic glial and glioneuronal tumors [20]. Promoter methylation and LOH without somatic mutations are known to downregulate *ATM *and *FHIT *genes in breast cancer [26,27]. It is reasonable to assume that the LOH due to deletion of one of the two alleles or due to loss of an entire chromosome with *TSC1 *or *TSC2 *gene in tumors will leave only one allele, resulting in the downregulation of these genes.

**Table 2:** Clinicopathological characteristics, LOH and gene expression variation in folds* for 16 tumor samples.

**Sample (Patient) no.**	**Age (yrs)/Sex**	**Tumor classification**	**Metastasis**	**Tobacco use**	**LOH@ *TSC1***	**LOH@ *TSC2***	***TSC1 (D)***	***TSC2 (D)***	***AKT1 (U)***	***PIK3C2A (U)***	***PDPK1 (U)***	***RHEB (U)***	***FRAP1 (U)***	***RPS6KB1 (U)***	***RPS6 (U)***	***EIF4E (U)***	***EIF4EBP1(D)***	***PTEN (D)***
54	40 M	T_4_N_1_M_0_	N/A	Yes	-	-	1.39	2.04	2.04	2.34	0.77	2.29	1.82	2.25	3.40	2.23	3.12	1.70
39	55 F	T_4_N_1_M_0_	Yes	Yes	-	-	1.77	1.95	4.04	2.47	2.33	1.92	2.68	3.34	2.67	3.01	0.98	1.44
19	48 F	T_4_N_2b_M_0_	No	Yes	-	-	1.93	3.57	3.29	1.63	1.57	2.12	2.21	2.93	2.01	2.07	1.45	2.04
15	55 F	T_3_N_1_M_0_	No	Yes	-	-	2.63	2.03	4.68	3.23	1.81	0.76	1.76	1.48	2.83	1.31	3.03	2.35
20	50 F	T_4_N_1_M_0_	Yes	Yes	-	-	1.98	2.03	1.46	1.65	0.65	0.85	0.92	1.34	1.50	1.98	3.02	1.87
8	50 F	T_3_N_1_M_0_	No	Yes	-	-	1.15	1.37	1.57	0.95	1.39	1.73	0.44	0.61	0.64	1.54	4.27	3.67
11	60 F	T_4_N_1_M_x_	Yes	Yes	-	-	0.31	0.69	1.80	1.69	1.03	2.54	3.76	1.94	0.69	1.17	0.96	2.36
40	50 F	T_2_N_1_M_0_	Yes	Yes	+	-	2.33	1.48	1.75	2.16	1.90	2.22	2.82	6.25	1.83	4.22	2.06	1.60
50	40 M	T_4_N_2b_M_0_	Yes	Yes	-	-	1.20	2.98	1.34	0.58	1.43	1.22	1.25	2.45	2.69	1.50	3.67	1.35
52	70 M	ED	N/A	No	-	-	2.35	1.82	2.69	2.64	1.50	2.23	5.78	1.80	1.76	3.74	1.69	2.23
32	35 M	T_4_N_0_M_x_	No	Yes	-	-	1.58	3.03	2.04	0.90	1.15	0.80	1.48	1.67	1.89	1.60	2.24	1.18
53	38 F	T_2_N_0_M_0_	No	Yes	+	-	7.92	1.51	3.68	2.88	2.59	2.78	1.19	2.32	1.21	2.13	2.77	1.99
55	40 F	T_4_N_1_M_0_	Yes	Yes	+	-	1.35	1.81	2.27	2.99	1.53	1.70	12.4	1.37	4.47	2.67	1.77	1.46
57	62 F	T_1_N_0_M_0_	Yes	Yes	-	-	1.40	2.39	2.26	4.50	8.26	2.95	2.24	5.05	1.80	2.32	2.17	0.50
59	70 F	T_4_N_2b_M_0_	Yes	Yes	-	+	2.38	1.30	1.48	2.80	1.92	1.67	1.38	0.67	1.66	1.70	5.07	1.23
60	40 F	T_4a_N_1_M_x_	Yes	Yes	-	-	1.37	1.80	0.87	2.28	1.49	1.98	1.05	1.04	1.99	1.88	0.60	1.30

* We defined the cutoff value for determining the upregulation or downregulation of a gene in a tumor sample as ≥ 1.8 fold difference in its expression between normal and tumor samples [3]. With this criterion, *TSC1*, *TSC2*, *EIF4EBP1 *and *PTEN *genes showed downregulation in 7/16, 11/16, 10/16 and 7/16 tumor samples, respectively. Whereas *AKT1*, *PIK3C2A*, *PDPK1*, *RHEB*, *FRAP1*, *RPS6KB1*, *RPS6 *and *EIF4E *showed upregulation in 10/16, 10/16, 6/16, 9/16, 8/16, 9/16, 10/16 and 10/16 tumor samples, respectively.

**Abbreviations**: TNM, Tumor, Node, Metastasis; and ED, Epithelial dysplasia.

'Yes' in tobacco use refers to addiction to tobacco, bidi and cigarettes for at least 15–20 years. '+' denotes LOH found; '-' denotes no LOH; N/A, not applicable;

(D)denotes downregulation of gene expression and (U)denotes upregulation of gene expression across 16 tumor samples.

**Figure 3:**
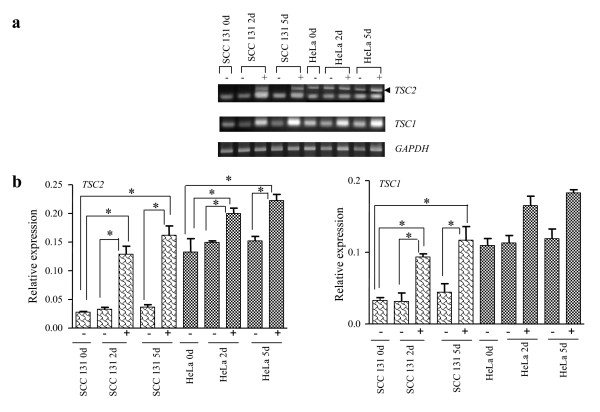
**Expression of TSC genes in cancer cell lines following treatment with 5-azacytidine for 2 (2 d) and 5 (5 d) days.** a) Representative RT-PCR gel pictures showing restoration or increase in the expression of TSC genes. b) Graphical representation of expression levels of *TSC1 *and *TSC2 *following the drug treatment. Expressions of these genes in untreated SCC 131 and HeLa cells at day 0, 2 and 5 were used as controls. '-' indicates no 5-azacytidine treatment and '+' indicates treatment with 5-azacytidine. Graphs represent mean ± SEM of two separate experiments. * indicates significant difference at p < 0.05. Although the expression of *TSC1 *was increased in HeLa cells following the drug treatment, the relative expression levels of treated and untreated cells were not significantly different from each other. The expression of *TSC2 *was significantly increased in both cell lines following the drug treatment.

Aberrant hypermethylation of promoter C_P_G islands has been found to be an important alternative mechanism to intragenic mutations for the inactivation of tumor suppressor genes [28]. Since we did not find any somatic mutation in TSC genes in oral tumors, we therefore ascribed the downregulation of TSC genes in oral cancer to an epigenetic alteration, resulting in the methylation of *TSC1 *and *TSC2 *promoters. To investigate if the downregulation of TSC genes in tumors is due to their promoters being methylated, we selected the oral cancer cell line SCC 131, which did not show expression of TSC2 and a low level of expression of TSC1 (Figure 1c), and treated it with methyltransferase inhibitor 5-azacytidine. RT-PCR data showed a significant increase in the expression of both the TSC genes in this cell line following the treatment (Figure 3). HeLa cells also showed a significant increase in the expression of *TSC2 *after 5-azacytidine treatment (Figure 3b). However, no significant difference was observed in the expression of *TSC1 *in HeLa cells following the drug treatment for 2 and 5 days (Figure 3b). No change in the expression of both the genes was seen in both cell lines grown for 2 and 5 days without the drug treatment (Figure 3b).

**Figure 4:**
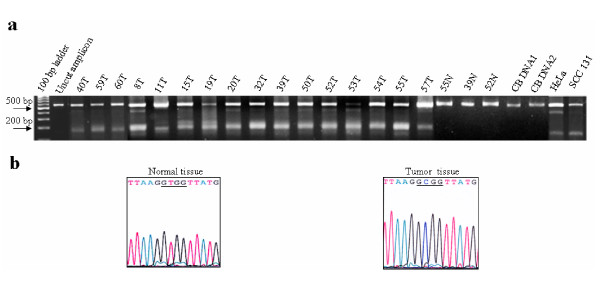
**Analysis of the methylation status of the *TSC2 *promoter by COBRA and bisulfite sequencing.** a) Bisulfite treated DNA samples from 16 oral tumors, three normal oral tissues, peripheral blood samples from two normal individuals and two cell lines were analyzed by COBRA. Normal and tumor samples are marked as N and T, respectively. Numbers correspond to patient numbers. Note, the undigested 571 bp fragment and a major digested fragment of ~175 bp in all tumors and cell lines. CB DNA1 and CB DNA 2 are peripheral blood DNA samples from two unrelated normal individuals. b) Representative bisulfite sequencing chromatograms of the promoter region from normal and tumor samples. The underlined region denotes the *Aci I *site which is lost in the normal tissue from patient 55 and retained in the tumor from patient 59 because of methylation at the C residue at this site.

In order to see if the downregulation of TSC genes is due to their promoters being methylated in tumors from the patients, we examined the methylation status of the promoters of both the TSC genes in a panel of 16 oral tumors, three normal oral tissues, two peripheral blood DNA samples from normal individuals, and two cell lines HeLa and SCC 131 by COBRA. Our repeated efforts to amplify the *TSC1 *promoter using different *Taq *DNA polymerases failed. We believe that this could be due to the nature of the sodium bisulfite treated DNA. However, we were able to successfully amplify the 571 bp long *TSC2 *promoter region. As can be seen from the Figure 4a, a 571 bp PCR product from all the tumors and both cell lines showed digestion, whereas PCR products from peripheral blood samples from two normal individuals and three normal oral tissues did not show digestion. Digested bands of varying sizes and intensities were seen in different tumor samples and both cell lines, in addition to a prominent digested band of ~175 bp and the uncut band of 571 bp (Figure 4a). In order to see if the digestion by *Aci I *was specific, we treated the PCR products with the restriction enzyme buffer only. The absence of any digestion suggested that the *Aci I *digestion of the samples was specific (data not shown). Sequence analysis of the PCR products from sodium bisulfite treated normal and tumor DNA showed that the treatment was specific (Figure 4b).

The variation in the degree of digestion by *Aci *I in different tumor samples might be accounted for by the fact that all surgical samples are likely to contain a heterogenous mix of normal and tumor cells as microdissection was not performed on tumor samples and also considerable heterogeneity of methylation might exist among tumor samples. An MSP (methylation-specific PCR) assay could not be designed in this study because the relevant methylated region was limited. Taken together, our 5-azacytidine and COBRA data suggested that TSC genes are targets of epigenetic inactivation in oral cancer (Figures 3 and 4).

### Aberrant expression of genes from the mTOR signaling pathway

**Figure 5:**
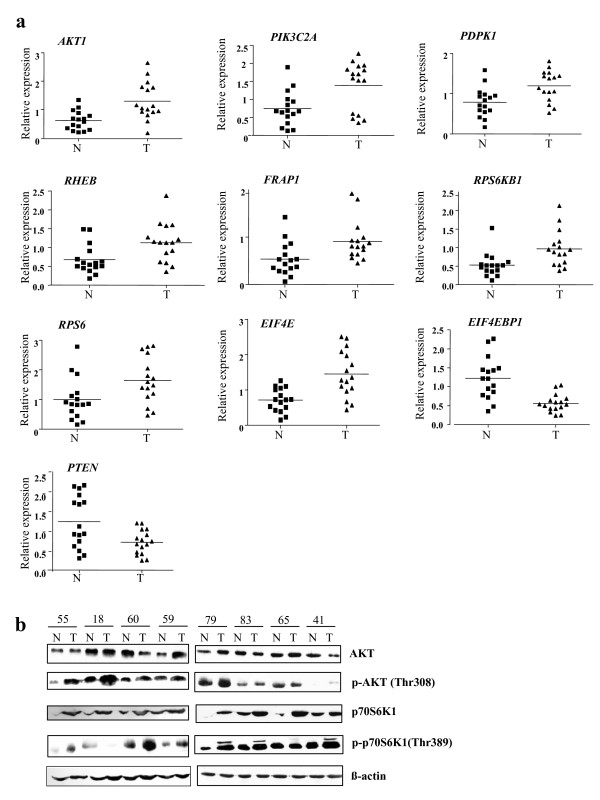
**Expression of other members of the mTOR signaling pathway in oral tumors.** a) mRNA expression of 10 genes in 16 matched normal and tumor samples. Horizontal lines represent mean values of mRNA expression across normal or tumor samples. b) Western blot analysis of matched normal (N) and tumor (T) samples from eight patients. Numbers represent patient numbers. Blots were probed with antibodies for anti-AKT, anti-p-AKT (Thr308), anti-p70S6K1 and anti-p-70S6K1 (Thr389).

Since TSC1 and TSC2 are important regulators of this pathway and showed downregulation, we hypothesized that other key players of this pathway might be also dysregulated in oral cancer. To this end, the expression pattern of other major regulators of this pathway [v-akt murine thymoma viral oncogene homolog 1 (*AKT1*); phosphoinositide-3-kinase, class 2, alpha polypeptide (*PIK3C2A*); 3-phosphoinositide dependent protein kinase-1 (*PDPK1*); Ras homolog enriched in brain (*RHEB*); FK506 binding protein 12-rapamycin associated protein 1 (*FRAP1*); ribosomal protein S6 kinase, 70 kDa, polypeptide 1 (*RPS6KB1*); ribosomal protein S6 (*RPS6*); eukaryotic translation initiation factor 4E (*EIF4E*); eukaryotic translation initiation factor 4E binding protein 1 (*EIF4EBP1*); phosphatase and tensin homolog, mutated in multiple advanced cancers 1 (*PTEN*); tyrosine 3-monooxygenase/tryptophan 5-monooxygenase activation protein, beta polypeptide (*YWHAB*); and insulin receptor substrate 1 (*IRS1*)] was investigated in the same panel of 16 matched normal and tumor tissues using semi-quantitative RT-PCR analysis. Mean expression levels of following genes showed significant upregulation in tumor samples: *AKT1 *(0.61 ± 0.08 in normal vs. 1.30 ± 0.16 in tumor, p = 0.0008), *PIK3C2A *(0.74 ± 0.12 in normal vs. 1.39 ± 0.17 in tumor, p = 0.0038), *PDPK1 *(0.79 ± 0.09 in normal vs. 1.19 ± 0.09 in tumor, p = 0.0041), *RHEB *(0.68 ± 0.09 in normal vs. 1.13 ± 0.13 in tumor, p = 0.0088), *FRAP1 *(0.54 ± 0.09 in normal vs. 0.91 ± 0.10 in tumor, p = 0.0095), *RPS6KB1 *(0.53 ± 0.08 in normal vs. 0.97 ± 0.12 in tumor, p = 0.0048), *RPS6 *(0.99 ± 0.18 in normal vs. 1.64 ± 0.19 in tumor, p = 0.02) and *EIF4E *(0.72 ± 0.08 in normal vs. 1.45 ± 0.17 in tumor, p = 0.0004) (Figure 5a). Whereas mean expression levels of *EIF4EBP1 *(1.23 ± 0.14 in normal vs 0.56 ± 0.06 in tumor, p = 0.0001) and *PTEN *(1.27 ± 0.17 in normal vs. 0.73 ± 0.08 in tumor, p = 0.0075) showed significant downregulation in tumor samples (Figure 5a). Two other genes of this pathway, *YWHAB *(0.81 ± 0.89 in normal vs. 1.06 ± 0.12 in tumor, p = 0.1153) and *IRS1 *(0.94 ± 0.10 in normal vs. 1.19 ± 0.12 in tumor, p = 0.1118) did not show any significant difference in expression levels between normal and tumor samples (data not shown). We then analyzed the expression pattern of a few genes (*AKT1 *and *RPS6KB1*) at the protein level using eight matched normal and tumor tissues. The level of total AKT1 was upregulated in 2/8 tumor samples only (Figure 5b). However, p-AKT1 (Thr308) showed upregulation in 7/8 samples (Figure 5b). p70S6K1 (RPS6KB1) and p-p70S6K1 (Thr389) were both upregulated in 8/8 and 6/8 tumor samples, respectively (Figure 5b). Increase in the phosphorylated forms of both AKT1 and its downstream effector p70S6K1 suggested an increase in their kinase activity, indicating a constitutive activation of this pathway in oral cancer.

Upregulation of *PIK3C2A *has been reported in several cancers such as cervical, colon, breast, liver, stomach and lung cancers [29]. Expression of this gene has been investigated in oral tongue carcinoma, and head and neck cancer cell lines where the mean expression level in tumor samples was found to be significantly higher than in normal samples [7]. Our results have also shown that *PIK3C2A *is upregulated in 10/16 oral tumors (Figure 5a, Table 2).

AKT1 (AKT) is a downstream effecter of PIK3C2A (PI3K). It has emerged as a central player controlling several signal transduction pathways that are activated in response to growth factors or insulin. Activation of AKT1 has been shown to be a frequent event in breast, colorectal, ovarian, pancreatic, and head and neck cancers [30]. One of the best characterized regulators of the mTOR signaling pathway is PTEN. The lipid phosphatase activity of PTEN acts as a negative regulator for PIK3C2A induced signaling as it dephosphorylates PIP3 [31]. PIP3 is a potent second messenger that recruits certain kinases to the plasma membrane including the Protein kinase B/Akt family of kinases and PDPK1. On membrane localization, AKT1 is activated in part through phosphorylation by PDPK1 and elicits several downstream cellular functions. Genetic inactivation of *PTEN *leads to constitutive activation of the mTOR pathway [32]. Our study has shown that *AKT1 *and *PDPK1 *show upregulation in 10/16 and 6/16 tumors respectively (Figure 5a, Table 2). *PTEN *on the other hand showed downregulation in 7/16 tumors (Figure 5a, Table 2). Mavros et al. [33] identified a low LOH rate of 12% in 50 samples of OSCC. They however did not find any mutation in the coding region of *PTEN *and concluded that the *PTEN *gene alterations do not play a key role in tumorigenesis of oral squamous cell cancers. We have also found a low LOH rate of 13% (6/46 informative cases) at the PTEN locus in the same panel of 50 paired blood and tumor DNA samples (data not shown). The frequency of LOH was 4.5% (2/44 informative cases) and 10% (3/30 informative cases) at D10S1765 and D10S541 respectively (data not shown). It is possible that the downregulation of this gene in oral tumors examined in this study is due to inactivating somatic mutations or its promoter methylation. However, these possibilities need to be investigated in the future.

FRAP1 has a central role in controlling cell cycle progression and cell growth. It has emerged as a major cancer therapeutic target [34]. FRAP1 exerts its effect by phosphorylating EIF4EBP1 (4E-BP1) which binds to and inactivates EIF4E, thus inhibiting 5'-cap-dependent mRNA translation. Phosphorylation of EIF4EBP1 releases EIF4E and allows initiation of translation. Regulation of EIF4E mediated translation is an important target for therapeutic intervention in light of the fact that *EIF4E *has been shown to be overexpressed in several cancers and that overexpression can cause malignant transformation of rodent fibroblasts [35]. In our study, *EIF4EBP1 *showed downregulation in 10/16 tumors (Figure 5a, Table 2). Upregulation of *EIF4E *in 10/16 tumors (Figure 5a, Table 2) potentiates its role in the increase of translation leading to overall cell growth and proliferation. FRAP1 also regulates translation via phosphorylation of a serine/threonine kinase p70S6K1 (RPS6KB1). Upon phosphorylation, p70S6K1 promotes translation of mRNAs containing a 5' terminal oligopyrimidine (5' TOP) by phosphorylating the ribosomal subunit S6. Since ribosomal proteins and translation elongation factors are encoded by 5' TOP mRNAs, signaling along the p70S6K1 pathway promotes ribosome biogenesis and overall protein biosynthetic capacity [4]. Our study provides the evidence that *FRAP1 *is upregulated in 8/16 tumors, as also are *RPS6KB1 *(9/16) and *RPS6 *(10/16) (Figure 5a, Table 2). Our western blot results also indicated that the *AKT1/RPS6KB1 *pathway is active in oral tumors, as phosphorylated forms of both proteins show increased levels in tumor samples (Figure 5b).

A novel positive regulator of FRAP1 is the small GTPase RHEB [4]. Tuberin/hamartin complex acts as a negative regulator of this pathway by C-terminal GAP activity of tuberin towards RHEB. When stimulated by growth factors, AKT1 relieves this inhibition by phosphorylation of tuberin, which dissociates the tuberin/hamartin complex [4]. Phosphorylated tuberin binds to the *14-3-3 *family of proteins which control various cellular functions [4]. We found upregulation of *RHEB *in 9/16 tumors (Figure 5a, Table 2), whereas we did not find any significant difference in the expressions of the tuberin interacting protein *YWHAB *(14-3-3β) and *IRS1 *across the samples analyzed (data not shown). Interestingly, 14-3-3zeta, another tuberin interacting protein [36], was recently found to be upregulated in OSCC [37]. Further, the expression of 14-3-3sigma, which also interacts with tuberin [36], was reduced or absent in OSCC [38]. This suggested that different isoforms of the 14-3-3 family behave differently in OSCC.

### Clinicopathological characteristics of patients with LOH at TSC1 and TSC2 loci

We correlated the presence or absence of LOH at the TSC1 and TSC2 loci in 50 primary tumors to their clinicopathological variables such as age, sex, T classification, stage, grade, histology, tobacco habits and lymph node metastasis. Fisher's exact test (two-sided) was carried out and a p < 0.05 was considered to be significant. Using the above criteria, none of the parameters examined demonstrated a significant correlation with LOH at either of the TSC loci (data not shown).

## Conclusion

Collectively, the detection of LOH in a proportion of OSCC samples coupled with reduced gene expression both at the RNA and protein levels indicates a loss of function of TSC genes, implicating their role as tumor suppressors in oral cancer for the first time. Loss of function of these genes may thus contribute to the constitutive activation of the mTOR signaling pathway leading to overall cell growth and proliferation. Our studies have also shown for the first time that several key members of this pathway show aberrant expression in oral cancer and can provide useful therapeutic targets. Several inhibitors of this pathway, such as rapamycin and its derivatives which inhibit mTOR (FRAP1) and the PI3K (PIK3C2A) inhibitor wortmannin, are in fact now being actively evaluated in clinical trials for other cancers [31,34]. Further, rapamycin and its derivative CCI-779 have been shown to reduce OSCC tumor size in nude mice [8,39]. Thus, these inhibitors could also be evaluated for the treatment of oral cancer.

## Competing interests

The authors declare that they have no competing interests.

## Authors' contributions

SC performed the experiments and provided inputs in drafting the manuscript. SMAM and KSG provided patient samples and analyzed clinical data. AK provided overall study design, guidance, drafted the manuscript and revision.

## Pre-publication history

The pre-publication history for this paper can be accessed here:


